# Efficient Vertical Structure Correlation and Power Line Inference

**DOI:** 10.3390/s24051686

**Published:** 2024-03-05

**Authors:** Paul Flanigen, Ella Atkins, Nadine Sarter

**Affiliations:** 1 Robotics Department, University of Michigan, Ann Arbor, MI 48109, USA; sarter@umich.edu; 2 Aerospace and Ocean Engineering Department, Virginia Tech, Blacksburg, VA 24061, USA; ematkins@vt.edu

**Keywords:** database, flight hazards, low-altitude flight, helicopter operations, advanced aerial mobility

## Abstract

High-resolution three-dimensional data from sensors such as LiDAR are sufficient to find power line towers and poles but do not reliably map relatively thin power lines. In addition, repeated detections of the same object can lead to confusion while data gaps ignore known obstacles. The slow or failed detection of low-salience vertical obstacles and associated wires is one of today’s leading causes of fatal helicopter accidents. This article presents a method to efficiently correlate vertical structure observations with existing databases and infer the presence of power lines. The method uses a spatial hash key which compares an observed tower location to potential existing tower locations using nested hash tables. When an observed tower is in the vicinity of an existing entry, the method correlates or distinguishes objects based on height and position. When applied to Delaware’s Digital Obstacle File, the average horizontal uncertainty decreased from 206 to 56 ft. The power line presence is inferred by automatically comparing the proportional spacing, height, and angle of tower sets based on the more accurate database. Over 87% of electrical transmission towers were correctly identified with no false negatives.

## 1. Introduction

Power lines are difficult to sense directly due to their small cross-section and irregular catenary shape. Occlusion from parallel wires, movement due to wind, sagging, and highly varied background further increase the power line sensing complexity. Tracking and indexing these structures relies on largely voluntary reporting followed by manual correlation and entry, which results in incomplete and inaccurate databases. The inadequate detection and perception of vertical structures is behind only a loss of situational awareness such as controlled flight into terrain (CFIT), as the most common known cause of fatal civilian helicopter accidents [1]. Obstacle strikes are also a leading cause of military rotor craft losses [2]. Wire strikes cause even more accidents than collisions with vertical structures [3] due to their near invisibility to the naked eye and sensors.

The paper proposes an automatic and efficient method to correlate sensed vertical structures. First, we create a hash table to enable lookup and correlation. Our hash key resolution is within DOF’s most accurate horizontal uncertainty, thus avoiding repeated entries for the same coordinates while retaining relevance throughout a hemisphere. Next, an index hash table (IHT) assigns this spatial key to existing entry values (such as position and height uncertainty). The uncertainty hash table (UHT) builds a list of vertical structure hashes whose uncertainty encompasses a given spatial hash. Observed towers are efficiently correlated and used to update existing IHT entries.

The refined IHT tower position is leveraged to infer the presence of associated power lines. Instead of searching for the virtually invisible wire, we predict a wire’s presence based on the configuration and geometric arrangement of the supporting transmission towers or other vertical structure. Pairs of towers that are within a distance proportional to their height and have a similar height are associated. The angle between continuous sets of three towers is also taken into account to reduce false positives.

This topic is of critical importance to low-flying aircraft ranging from the helicopters of today to the advanced air mobility (AAM) and uncrewed aircraft systems (UAS) of tomorrow.

The goals of this article are to first efficiently consolidate vertical structure position information from sensor data and then find power lines based on structure locations. This article offers a method for efficiently correlating and updating existing vertical obstacle databases with new observations. Next, towers within the updated database are compared to infer the presence of power lines. Specific contributions are as follows:

- A new method to efficiently correlate vertical structures;
- A novel approach to reliably finding potentially hazardous wires based on their arrangement, proximity, and similarity;
- Evaluation of these approaches against the current Delaware Digital Obstacle File.

Following the background and literature review, Section 2 provides an overview of the database update and power line inference methods. Section 3 describes the FAA Digital Obstacle File used for our experimentation. Section 4 presents results of our methods. Section 5 discusses the overall performance of our method, while Section 6 offers conclusions.

### 1.1. Current Uses of Vertical Structure Data

There are several examples where large amounts of raw information is organized into useful maps. This integrated data can then inform motion planning or other forms of action selection and decision making.

Several approaches use pole mapping for vehicle localization. The large data scale requires that the area of consideration be down-sampled due to onboard computer system storage and computation time constraints. Detecting numerous poles that maintain the same physical characteristics over time provides an opportunity to match detected pole patterns with an a priori map [4]. The technique in [4] used quantization to compare an average of 18 poles within 50 m of the moving vehicle with an a priori pole map with one-meter accuracy to locate a vehicle’s position. A more recent mapping approach minimizes residual error between extracted pole and road curb points in successive frames to create a local feature map [5]. Neither of these techniques update the high-accuracy a priori pole map. Both approaches rely on high resolution at close range for efficient correlation. Refs. [6,7] project extracted points to the ground plane as squares. If the squares overlap, they are combined into a single-pole entity. Poles that are not seen repeatedly are removed from the map using this sliding window. The extracted poles are compared with a 30 m square reference map for localization. Recent approaches that use semantic labeling for localization and mapping rely on continuous, dense surfaces that are associated with rich imagery [8].

**Figure 1:**
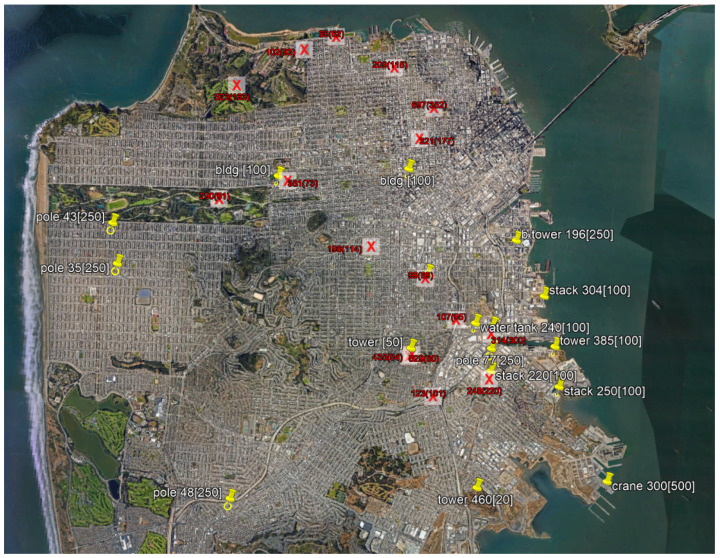
Vertical obstacles in San Francisco. Towers and other vertical structures from DOF are shown with yellow pins. Obstacles from DVOF are denoted by red X’s.

Above the ground level, the Federal Aviation Administration’s (FAA’s) Digital Obstacle File (DOF) is the definitive, publicly available source for vertical structures that could be a hazard to flight operations. Currently, adding and revising human-made obstacles that are far away from airports with instrument approaches largely relies on voluntary reporting from infrastructure builders. These reports are occasionally supplemented by imprecise observations from aircrews or ground personnel. Reported vertical structures are manually entered into the database and are seldom confirmed by onsite inspections or correlation with other data sources. High-resolution 3D data, such as LiDAR point clouds, is not allowed to be a primary source of obstacle information [9]. Obstructions greater than three miles from designated airports that are less than 499 ft above the ground level are not considered “obstructions to air navigation”, ref. [10] making low-altitude obstacles especially prone to oversight. Figure 1 shows an example of the disparity in two leading vertical obstacle data bases: the Federal Aviation Administration’s (FAA) Digital Obstacle File (DOF) and the National Geospatial-Intelligence Agency’s (NGA) Digital Vertical Obstacle File (DVOF). Although some obstacles are mutually represented, a significant number of vertical structures are present in only one of the two databases.

DVOF and DOF use cases focus on two domains: ground-level and flight altitudes greater than 500 ft above ground level. These focus areas omit the low-altitude flight environment that is essential for traditional helicopters, emerging advanced aerial mobility (AAM) aircraft, and small uncrewed aircraft systems (UASs).

### 1.2. Current Data Structures

Efficiently representing the environment is a long-standing challenge. The data structure for the ground-level domain supports either short-range forecasting or high-level exhaustive comparison. Ref. [4] used quantization that divided the area of consideration based on radius (50 m) and position certainty (1 m). Infrastructure predictive mapping [11] consolidated the structures along with other map features and tiles in the GeoPackage data structure [12].

The data structure for aerial obstacles must include large geographical areas, but data latency depends on manual examination and verification. Obstacle accuracy and completeness wane at lower altitudes away from major airports. In DOF, an accuracy better than 250 ft horizontally and 50 ft vertically is only motivated by the need for obstacle clearance when descending to or departing from airports [13]. As a result, over half of the DOF obstacles have a position uncertainty larger than 250 ft horizontally and 50 ft vertically.

Other work has found ways to efficiently map more general features in other safety-critical applications. Recent approaches use feature matching between previously gathered point clouds and current perspectives [14]. Although this approach minimizes the memory required to match features in a variety of locations, it relies on a pre-processed, high-resolution point cloud previously gathered from a viewpoint that is similar to the current 3D sensor perspective.

### 1.3. Power Line Mapping

Due to the ever-changing nature and sheer quantity of electrical infrastructure, there have been several efforts to automate power line mapping. A crowd-sourcing approach [15] has been proposed but is no longer online. Recent efforts use night-time lighting patterns in satellite imagery to predict the infrastructure position to within 1000 m 70% [16] or 75% [11] of the time.

Other wire-finding methods depend on detecting wires directly. Airborne methods that automatically segment power lines rely on continuous contact [17,18], known location [19,20], and/or very close range [21,22,23]. We propose to leverage the fact that power lines and other wires are intrinsically associated with more apparent vertical structures.

### 1.4. Problem Statement

Quickly consolidating ever evolving information is the leading challenge. The previously described automatic approaches are vulnerable to false positives and false negatives. Given multiple incomplete and inaccurate databases and current observations, how can obstacles be reliably and automatically correlated and updated? Given the additional position accuracy, how can tower arrangement and relationship be used to infer the presence of power lines?

## 2. Methods

**Figure 2:**
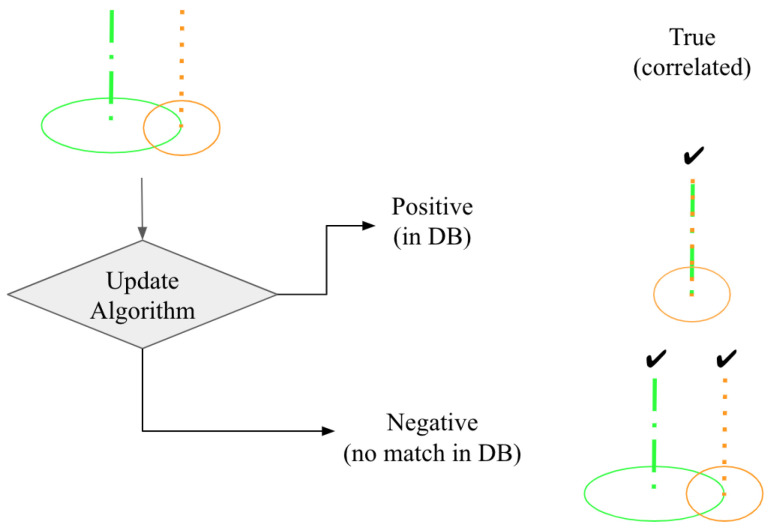
Tower correlation options. Algorithm 1 compares an existing vertical structure entry and associated horizontal uncertainty (green) with an observation (orange). The observation and entries are either correlated and consolidated (true positive) or the observation is found to not exist in the database (true negative).

Our previous work presented methods for efficiently locating prominent vertical structures in large-point cloud data. We will compare these precise positions with the known a priori obstacle databases to create a more comprehensive and complete obstacle listing. Figure 2 presents a proposed method for correlating detected vertical structures to an existing database. If the vertical structure is not within the horizontal uncertainty of an existing structure, it will be added to the database as a new item. If the detected structure is within the horizontal uncertainty of a structure described in the database, they will be correlated and the database meta data will retain the more accurate position attributes.

Next, Section 2.2 uses this refined vertical obstacle position database to infer the presence of power lines. This method starts by associating vertical structures that have a similar height and proportional distance using Algorithm 2. Then, Algorithm 3 iterates through each set of vertical structures, checking whether the interior angle exceeds pre-determined alignment criteria.
**Algorithm 1**: Update algorithm. Array of existing database entries represented with prior subscript. Array of aspects of newly observed structure denoted by obs subscript.1:**procedure** UpdateExisting(IHT,UHT,Obs)2:    $LatLonHash\leftarrow SpatialHasher(lat_{obs},lon_{obs})$3:    **if** UHT[LatLonHash] **then**                 ▹ if observed hash exists in prior4:        **for** center∈UHT[LatLonHash] **do**5:           prior←IHT[center]6:           $Upper_{prior}\leftarrow Ht_{prior}+VertUncert_{prior}$7:           $Lower_{prior}\leftarrow Ht_{prior}-VertUncert_{prior}$8:           **if** obsHt≥priorLower,obsHt≤priorUpper **then**             ▹ similar height9:               $horiDist\leftarrow distBetween(lat_{prior},lon_{prior},lat_{obs},lon_{obs})$10:               $age_{prior}\leftarrow \Delta date_{prior}$11:               $age_{obs}\leftarrow \Delta date_{obs}$12:               $age_{weight}\leftarrow \frac{age_{obs}}{age_{obs}+age_{prior}}$13:               **if** $horiUncert_{obs}\leq horiDist$ **then**14:                   $horiDist\leftarrow horiUncert_{obs}$15:               $horiDisp\leftarrow age_{weight}\cdot horiDist$16:               $latLon_{update}\leftarrow pointOffset\left(horiDisp,lat_{prior},lon_{prior},lat_{obs},lon_{obs}\right)$17:               **if** $horiUncert_{obs}\leq horiUncert_{prior}$ **then**18:                   $horiUncert_{update}\leftarrow horiUncert_{obs}$19:               **else**20:                   $horiUncert_{update}\leftarrow horiUncert_{prior}$

**Algorithm 2**: Tower association algorithm where ϵ represents the height uncertainty and PL is a list of sublists of tower spatial hash keys.
1:ν← height–distance ratio2:α← additional height buffer3:$z_{upper}\leftarrow$ upper-height limit4:$z_{lower}\leftarrow$ lower-height limit5:**procedure** ListBuilder(obs,IHT)6:    **for** i∈obs **do**7:        $obs_{key}\leftarrow SpatialHasher\left(obs\left(i\right)\right)$8:        $Dist_{max}\leftarrow obs{\left(i\right)}_{z}\cdot \nu$9:        $z_{max}\leftarrow obs{\left(i\right)}_{z}+obs{\left(i\right)}_{\epsilon }+\alpha$10:        $z_{min}\leftarrow obs{\left(i\right)}_{z}-obs{\left(i\right)}_{\epsilon }-\alpha$11:        **if** $obs{\left(i\right)}_{z}>z_{upper}$ AND $obs{\left(i\right)}_{z}<z_{lower}$ **then**12:           **for** j∈IHT**do**                      ▹ search subset in vicinity13:               Dist←distanceBetween(obs(i),IHT(j))14:               **if** $Dist<Dist_{max}$ **then**15:                   **if** $IHT{\left(j\right)}_{z}\geq z_{min}$ AND $IHT{\left(j\right)}_{z}\leq z_{max}$ **then**16:                       found←False17:                       **for** k∈PL**do**                        ▹ k = sublist18:                          **if** $obs_{key}\in k$ **then**           ▹ if observed tower exists on sublist19:                              $PL\left(k\right)\leftarrow IHT{\left(j\right)}_{key}$            ▹ add similar tower to sublist20:                              found←True break21:                          **if** $IHT{\left(j\right)}_{key}\in k$ **then**             ▹ if similar tower is on sublist22:                              $PL\left(k\right)\leftarrow obs_{key}$               ▹ add observed tower to sublist23:                              found←True break24:                          **if** not found **then**          ▹create new sublist with both towers25:                              $PL\leftarrow [obs_{key},IHT\left(j)\right]$


**Algorithm 3**: Transmission tower list checking algorithm. PL= list of sublists, *L*, of tower spatial hash keys. Additional alignment checks in blue.
1:n← minimum number of towers per list2:$\theta_{min}\leftarrow$ minimum tower angle3:$\theta_{align}\leftarrow$ maximum angle for alignment4:$Ht_{max}\leftarrow$ maximum height for less-aligned series5:$HDR_{series}\leftarrow$ height–distance ratio for less-aligned series6:**procedure** ListScrubber(PL)7:    **for** L∈PL **do**8:        **if** length(L)≥n **then**9:           L←sorted(L)                    ▹ order entries W-E, N-S10:           **for** i∈length(L−2) **do**11:               $T_{1}\leftarrow L\left(i\right)$12:               $T_{2}\leftarrow L\left(i+1\right)$13:               $T_{3}\leftarrow L\left(i+2\right)$14:               $\theta_{series}\leftarrow 180-angleBetween\left(T_{1},T_{2},T_{3}\right)$15:               $Ht_{series}\leftarrow maxHeightBetween\left(T_{1},T_{2},T_{3}\right)$16:               $Dist_{series}\leftarrow maxDistBetween\left(T_{1},T_{2},T_{3}\right)$17:               $Dist_{max}\leftarrow maxHt\left(T_{1},T_{2},T_{3}\right)\cdot HDR_{series}$18:               **if** i == 0 **then**19:                   **if** $\theta_{series}<\theta_{align}$ **then** continue20:                   **if** $\theta_{series}>\theta_{min}$ **then** continue    ▹ no continue for alignment checks21:                       **if** $Ht_{series}<Ht_{max}$ **then** continue22:                       **if** $Dist_{series}<Dist_{max}$ **then** continue23:                       **else**PL.append(L[i+1:])24:                   **else**PL.append(L[i+1:])            ▹ add new *L* without first tower25:               **else**26:                   **if** $\theta_{series}<\theta_{align}$ **then** continue27:                   **if** $\theta_{series}<\theta_{min}$ **then** continue28:                     **if** $Ht_{series}<Ht_{max}$ **then** continue29:                     **if** $Dist_{series}<Dist_{max}$ **then** continue30:                     **else**PL.append(L[i:])31:                   **else**PL.append(L[i:])


### 2.1. Efficient Database Updates

We create a hash table to enable lookup and correlation with constant time complexity. Hashtables rely on a succinct and descriptive key. Our key is based on the current World Geodetic System (WGS) 1984 projection. Selecting a resolution of 0.25 s equates to approximately 25 ft in latitude and between 19 and 24 ft in longitude for the continental United States. This resolution is within DOF’s most accurate horizontal uncertainty, thus avoiding repeated entries for the same coordinates.

In degree, minute, and second format, representing latitude or longitude requires seven digits (DD MM SS.SS) when we remove the character specifying the hemisphere. We retain degrees to retain relevance throughout a hemisphere. Also, a single degree of longitude is only 48 miles at the northern section of the continental United States, which is well within the span of an aerial vehicle’s operating range. Combining latitude and longitude provides a unique 16 character string.

Next, we build an index hash table (IHT). This table reads in the data fields from an existing DOF or DVOF database and adds a spatial hash key based on rounding the given coordinates up to the nearest 0.25 s. Simply searching for the nearest neighbor is not sufficient due to numerous vertical obstacles and their associated large, uneven, and overlapping horizontal uncertainties. An updated vertical structure coordinate may not be closest to its existing database location. Exhaustively searching within the overlapping uncertainties (even with the reduced spatial hash resolution) requires checking at least 9 (for 20 ft horizontal uncertainty) and rapidly increasing to over 54,000 cells with a horizontal uncertainty of one nautical mile. Given that most obstacles in DOF have an uncertainty of at least 250 ft (143 cells), the search process must be efficient.

**Figure 3:**
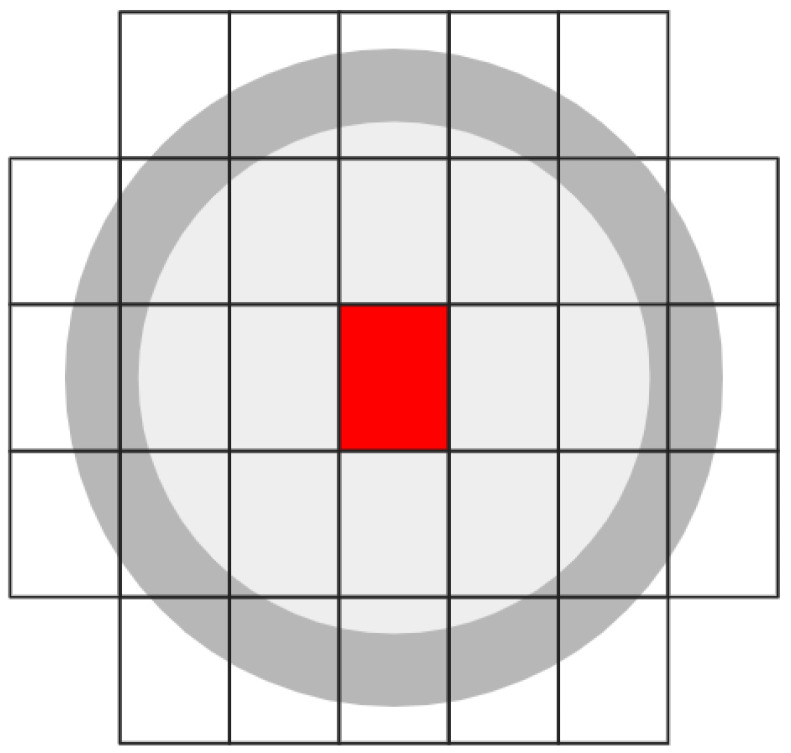
The light gray inner circle has a 50 ft radius to represent 50 ft horizontal uncertainty. The darker gray boundary is thickened to represent imprecision. This allows the center to be anywhere within the red center spatial hash’s 0.25 s resolution. The 30 outlying cells encompass this uncertain circumference.

We use the IHT with spatial hash keys to create a second hash table. The purpose of this second hash table is to readily determine whether a queried location is within the horizontal uncertainty of an existing vertical object. The uncertainty hash table (UHT) builds a list of vertical structure hashes whose uncertainty encompasses a given spatial hash. For example, the spatial hash for the center red cell in Figure 3 would be the value for the 31 keys that are the spatial hash for each of the cells that encompass the provided horizontal uncertainty.

**Figure 4:**
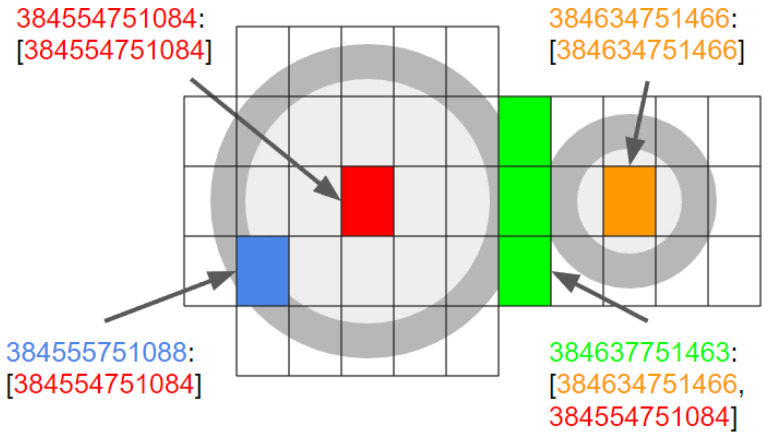
Three cells that coincide with 50 ft (left) and 25 ft (right) horizontal uncertainties are shown in green. In the uncertainty hash table (UHT), the entries for the red and orange center cells of the 50 and 25 ft circles have the same spatial hash for the key and value. The off-center blue cell UHT entry has the blue spatial hash for its key, but the corresponding value is the spatial hash for the center coordinate of the 50 ft circle. Each green cell has its coordinate’s spatial hash for the key, but because the cells are within the uncertainty of two vertical structure entries, each green key has two values.

When the array of uncertainty cells for an entry in the IHT overlap with another, as they do in Figure 4, the UHT key includes values for each relevant index. We instantiate a multimap to allow multiple entries for each key in the UHT.

**Figure 5:**
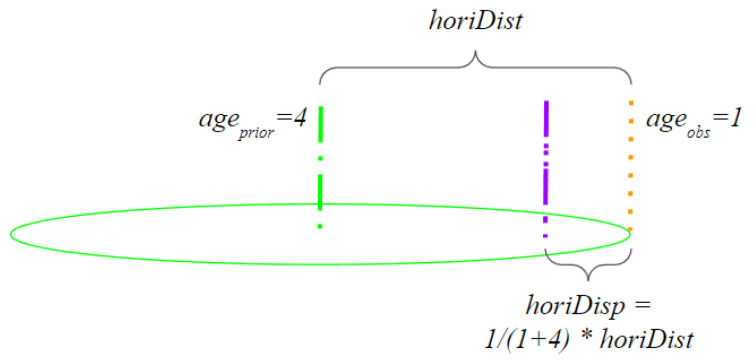
Example of position update procedure based on age of observation. The prior four-year-old tower observation (green) is compared to the more recent one-year-old tower observation (orange). The updated position (purple) is 1/5 of the horizontal distance between the prior entry and the observation.

After creating the initial IHT and the corresponding UHT, we incorporate new observations. An observation consists of the latitude, longitude, height above ground level (AGL), horizontal uncertainty, and observation date. Algorithm 1 provides an overview of the update process. If an observed vertical structure’s coordinate exists within the UHT, the observation is compared to each IHT element in the UHT list. First, we determine that the observation and existing IHT entry are similar when the observed height is within the uncertainty range of the existing entry. If the heights coincide, we determine the horizontal distance between the observation and the prior entry. Instead of updating the entry to the midpoint, we place the new location based on the age of the observation (Equation (1)). Since the majority of DOF entries are many years old, we make displacement proportional to the relative age of the observation and the prior database entry, as shown in the example in Figure 5. The horizontal uncertainty is updated to the observed horizontal uncertainty as long as the observed uncertainty is less than the prior IHT entry.

(1) $$horiDisp=\frac{age_{obs}}{age_{obs}+age_{prior}}\cdot horiDist$$

### 2.2. Power Line Inference

Using our more accurate and comprehensive vertical structure catalogs, we then implement and evaluate a power line finding algorithm.

We hypothesize that at least three towers are necessary to support power lines and other wires. We also hypothesize that a line of at least three power line pylons would share three characteristics:

1. The height above ground for each tower will be within a certain range;
2. The angle between this set of towers will be less than 90 degrees;
3. The spacing between successive towers will be within a certain range.

**Figure 6:**
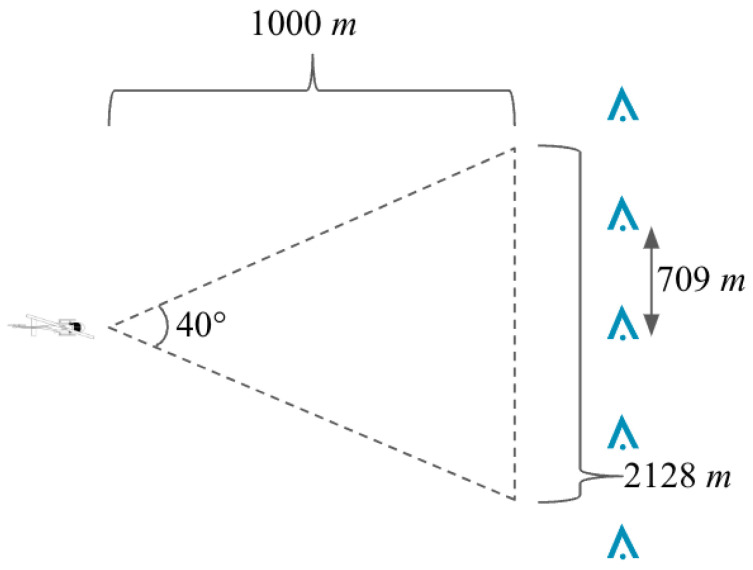
Top–down view of a notional airborne 3D sensor (not to scale). With a 1000 m sensor range and 40° azimuth, at least three vertical obstacles (shown in blue) with spacing ≤ 709 m will be present in the field of view.

We assume a tower detection range of 1000 m. This conservative detection range coupled with a sensor azimuth of 40° could encompass three transmission towers supporting power lines with over 700 m uniform spacing ($\frac{2128\;m}{3towers}$) as shown in Figure 6. This wide field of consideration is also suited for the low-altitude flight profile where the heading and flight path is consistently varied. The approach equally considers obstacles across the entire field of view versus depending on narrow foveal vision. Although this approach would be designed for relatively simple online implementation, it could function just as well in an offline application where the power line presence is inferred prior to takeoff.

Our approach starts by associating towers with a similar height and within a certain distance. Distance between towers is proportional to the tower height. In addition to tower height, the upper limit ($z_{upper}$) is defined by the uncertainty of the vertical measurement plus a global additional height buffer, α.

**Figure 7:**
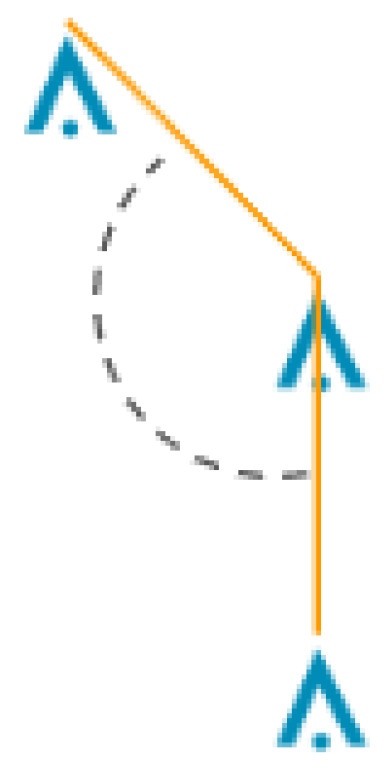
Top–down view of the interior dashed angle between three towers supporting an orange power line.

Next, Algorithm 3 cycles through each list of associated towers, *L*. It starts by sorting the list entries so that they are arranged from West to East, then North to South. Then, it removes any tower list that contains less than three towers. Next, the algorithm checks whether the interior angle between each set of three towers in the list has an interior angle greater than or equal to the minimum tower angle, $\theta_{min}$. Figure 7 shows an example of an interior angle.

Algorithm 3 also includes provisions for a more nuanced angle-based check. The blue text shows a secondary level of more restrictive height difference and distance if the towers are not aligned within a more conservative $\theta_{align}$. This secondary approach is designed to be more permissive for closely aligned towers while maintaining scrutiny on towers that are not in a straight line.

## 3. Setting

**Figure 8:**
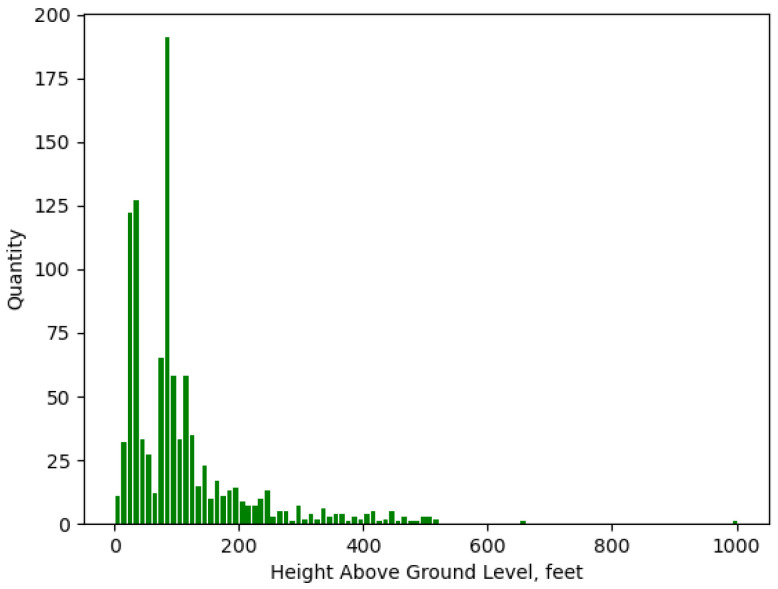
Histogram for vertical obstacles contained in Delaware’s Digital Obstacle File.

We use the Federal Aviation Administration’s publicly available Digital Obstacle File (DOF) as a baseline. DOF contains multiple descriptive fields for each obstacle entry, including location, height AGL, height accuracy, horizontal accuracy, and revision date. DOF files are separated by state and issued every 56 days. For our subsequent analysis, we chose the DOF file for the state of Delaware. Delaware offers a variety of urban, suburban, and rural infrastructure in a compact dataset. A histogram of Delaware’s 19 March 2023 DOF is shown in Figure 8. Table 1 shows the breakdown of horizontal accuracy attributed to each DOF entry. Of the 1063 objects, more than half have a horizontal uncertainty greater than 250 ft with an overall average horizontal accuracy of 206 ft. On average, the last time a DOF entry was added, updated, or verified was 2015.

The National Geospatial-Intelligence Agency has a similar vertical obstacle database, known as the Digital Vertical Obstacle File (DVOF). This database is unclassified but only available to United States government employees. For the state of Delaware, DVOF contains over 15,000 entries with an average horizontal accuracy of less than 100 ft. For this analysis, we use Delaware’s DVOF as observations against DOF.

## 4. Results

Delaware’s DOF dataset offers a variety of vertical obstacles. First, we ingest the DOF information into an index hash table (IHT) and assign unique spatial hash values. Next, we build an uncertainty hash table (UHT) which populates spatial hash cells within the radius of the existing DOF coordinate. This evaluation then finds whether a more recent DVOF observation lies within the UHT and proceeds to correlate and update the original DOF entry. We use the improved database for power line inference.

### 4.1. Database Updating

**Figure 9:**
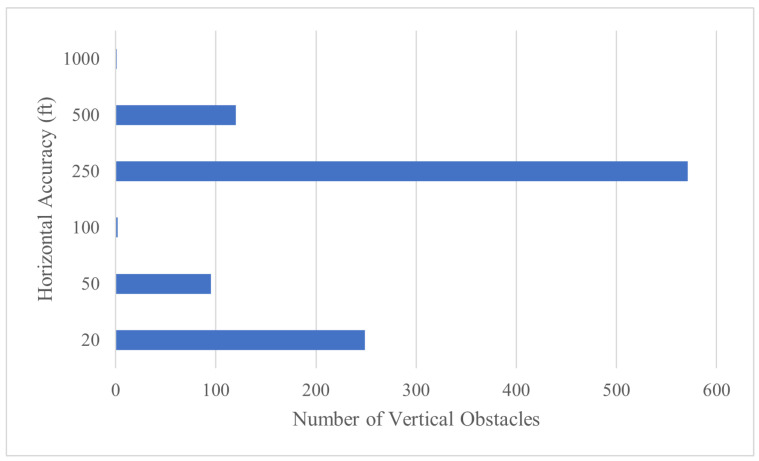
Original distribution of Delaware DOF horizontal uncertainty.

Figure 9 shows that, before implementing DVOF observations with Algorithm 1, the most frequent horizontal uncertainty value was 250 ft. The 1063 entries had an average horizontal accuracy of 206 ft.

**Figure 10:**
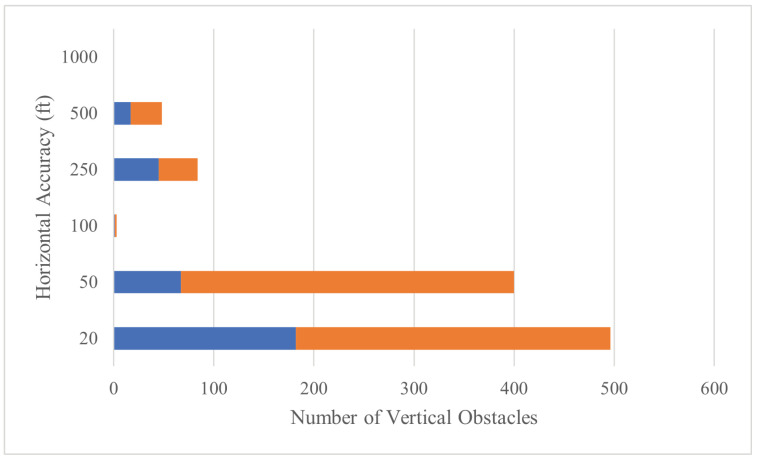
Distribution of Delaware horizontal uncertainty after correlation. Blue bars represent entries that were not changed, orange bars represent updated entries.

Figure 10 shows (in orange) how 719 of the original 1063 entries were updated. Including the 344 uncorrelated entries (in blue), the overall horizontal uncertainty decreased to 56 ft.

We apply our method to all vertical structure entries in the refined list that result from the previously described database updating approach. This exhaustive comparison provides a sense of effectiveness with a variety of transmission tower arrangements. An entry in the Delaware DOF that was in the transmission line tower category and was also inferred to be a transmission line tower was a true positive (TP). An entry that was correctly categorized as another type was a true negative (TN). False positives (FPs) were DOF entries that were erroneously categorized as a transmission tower. False negatives (FN) were entries which were categorized as transmission towers in the DOF database, but were not labeled as a transmission tower with the previously described methods.

Table 2 categorizes several existing database update methods. These data structures either compile (in that they add new observations) or match (in that they quickly aggregate new obstacle information along with uncertainty). No existing approach both automatically (as opposed to manually) evaluates new entries and supplements (as opposed to overwriting) existing entries. Our proposed method refines object position accuracy, automatically adds new entries, and automatically supplements existing database objects.

### 4.2. Power Line Inference

#### 4.2.1. Rejecting False Negatives

The most essential aspect while deducing the presence of power lines is avoiding false negatives. This requires the tower assembled transmission tower lists to include all potential transmission towers. The upper- and lower-height limit ($z_{upper}$ and $z_{lower}$) in Algorithm 2 were set to 300 and 49 ft, respectively, according to descriptions of recent transmission tower construction projects [24,25]. Height–distance ratio, ν, was evaluated from 1 to 15. The additional height buffer, α, was evaluated from 5 to 200 ft in 5 ft increments.

**Figure 11:**
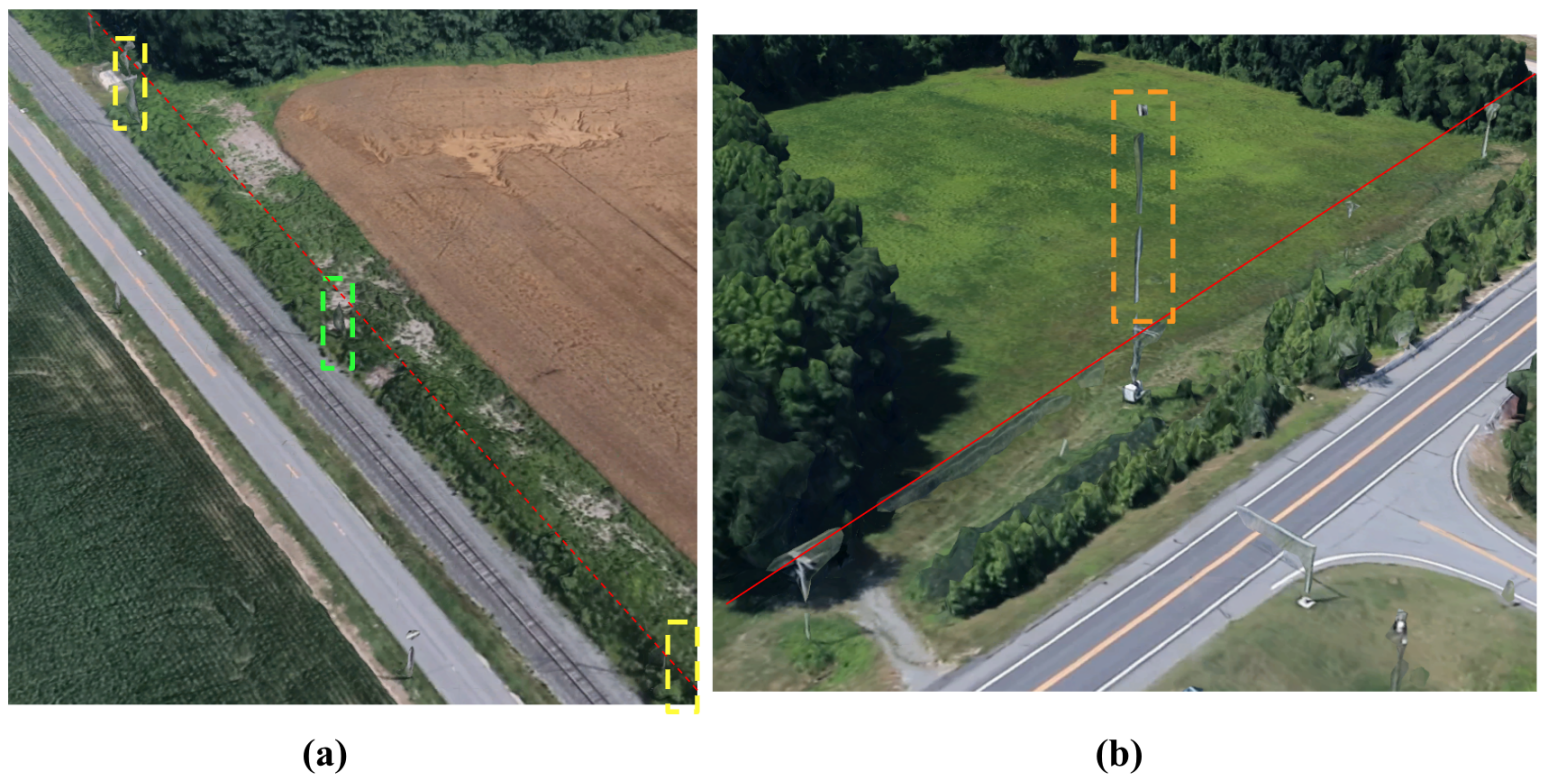
Isolated exceptions in the Delaware DOF. The 68 ft tall tower in the green rectangle (**a**) is within 230 ft of the accompanying towers in yellow rectangles that support the dashed red power line. A 123 ft cell tower (dashed orange rectangle) co-located with a line of uncharted 30 ft utility poles supporting the red power line (**b**).

**Figure 12:**
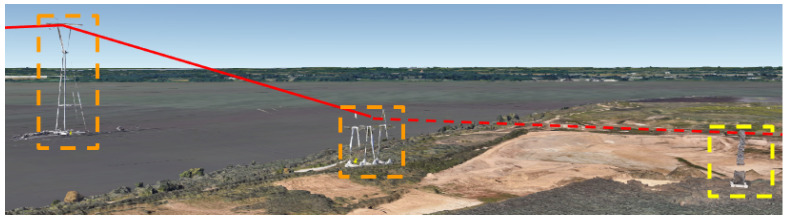
A tall 433 ft cell tower in the Delaware river (left dashed orange rectangle) is much higher than the 139 ft tower on the shoreline. The next land-based tower (yellow dashed rectangle) does not exist in Delaware’s DOF.

There were several examples of entries in the Delaware DOF that were categorized as transmission line towers that escaped the consolidation approach in Algorithm 2. Most of these exceptions were due to isolated entries which only show one or two transmission towers of a much longer series (Figure 11). Other transmission towers had significant height variations (Figure 11 and Figure 12). Isolated entries were removed from further analysis since associated towers did not exist in Delaware’s DOF.

**Figure 13:**
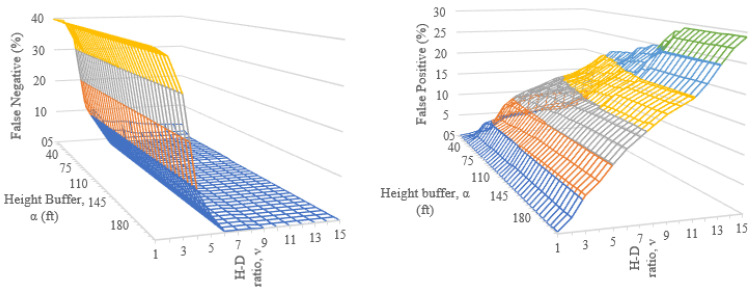
Results fromevaluating tower association algorithm, false negative (**left**) and false positive (**right**).

Figure 13 shows how false positives increase with a larger height buffer and height–distance ratio. False negatives show the opposite trend, dropping sharply with a height–distance ratio, ν, greater than 6. When ν=85ft and α=9, the false negative rate is zero and the false positive rate is 14.9%.

#### 4.2.2. Further Reduction in False Positives

**Figure 14:**
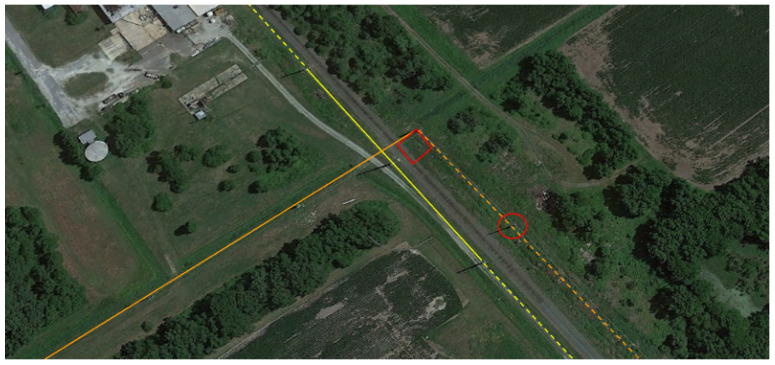
A line of charted towers supports an solid orange power line which takes a 90 degree turn at the tower in the red square. The tower in the red circle is rejected due to this sharp angle. Subsequent towers supporting the same power line (now a dashed yellow line) are not charted. Three towers in the Delaware DOF support a power line shown with a solid yellow line; the towers supporting the dashed yellow portion are not charted.

**Figure 15:**
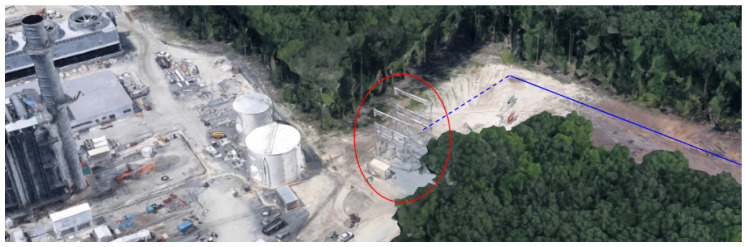
A transmission tower (red circle) is not categorized as a transmission tower supporting the dashed blue power line due to the sharp 90 degree turn towards the power plant. The power plant’s 189 ft smokestack is an entry in Delaware DOF.

**Figure 16:**
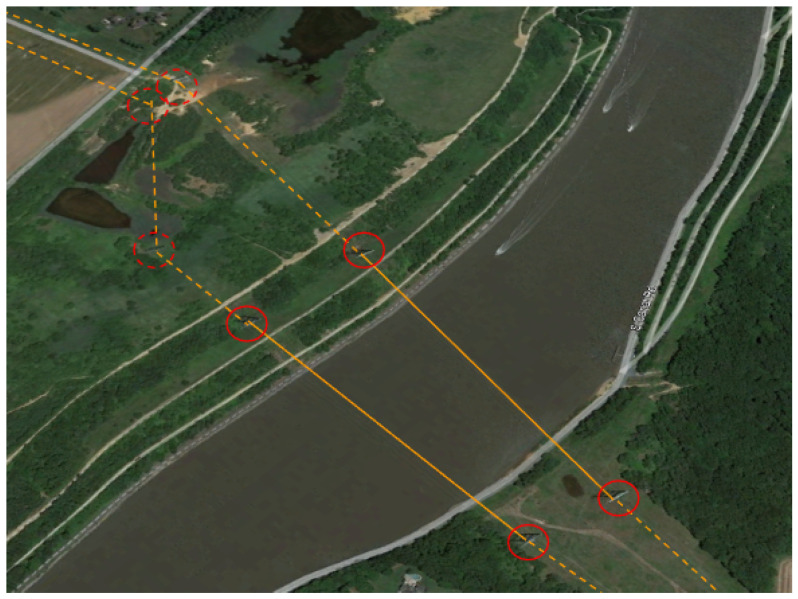
Four transmission towers (solid red circles) that support a power line (orange line) across a river are in the Delaware DOF and are correctly recognized by the list-builder algorithm. The angle-checking algorithm rejects the assignment due to their rectangular arrangement. Uncharted towers (dashed red circles) that support the terrestrial portion of the power lines (orange-dashed lines) are not in the Delaware DOF.

We hypothesized that false positives would reduce when successive sets of three towers within a list *L* were closely aligned with a large interior angle. Continuing with ν=85ft and α=9, the minimum tower angle, $\theta_{min}$ was incrementally evaluated according to the basic (black only) steps in Algorithm 3. Even with a conservative 90 degree angle between towers, the algorithm isolated and incorrectly rejected several entries that were categorized as transmission towers. Figure 14 shows an example of a tower (in red circle) that is rejected. If at least two more towers supporting the same dashed orange power line were charted, the span would be depicted as a separate span. Figure 15 is an example of a single tower at the beginning of an electrical transmission line. Figure 16 shows another example of towers that are not in the Delaware DOF past the shoreline.

**Figure 17:**
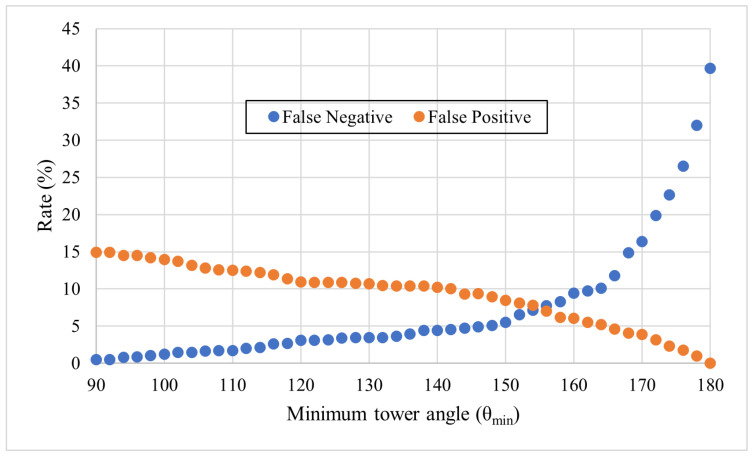
Concentration of false positive and false negative tower assignments with varying minimum tower angles, $\theta_{min}$.

Even by ignoring these isolated entries, false negatives grew significantly while increasing the minimum tower angle, $\theta_{min}$ (Figure 17). False positives slowly decreased with increasing $\theta_{min}$.

**Figure 18:**
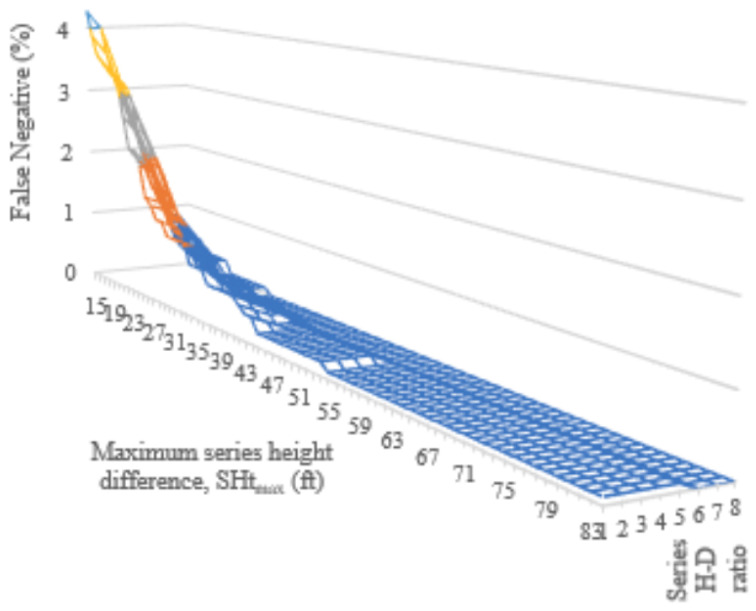
False negative rates obtained by the incremental checking of maximum series height differences ($SHt_{max}$) from 15 to 84 ft and height–distance ratios from 1 to 8.

**Figure 19:**
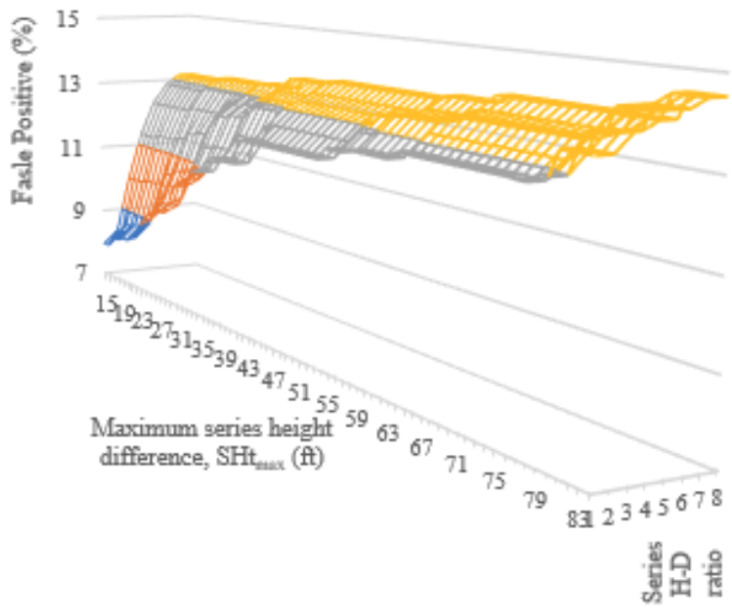
False positive rates obtained by the incremental checking of maximum series height differences ($SHt_{max}$) from 15 to 84 ft and height–distance ratios from 1 to 8.

This rising number of false negatives led to the implementation of the secondary alignment checks in Algorithm 3. The secondary checks applied to sets of three tower series that had an angle of alignment, $\theta_{align}$, less than 4 deg. False negative and false positive rates for maximum series height differences ($Ht_{max}$) and height–distance ratios that were more restrictive than the baseline ν=85ft and α=9 resulted in Figure 18 and Figure 19. As with the previous incremental checks, false negative rates increased when a smaller allowable height difference occurred between the towers. False negative rates also increase with a smaller height–distance ratio, $HDR_{series}$. False positive rates showed the opposite tendency, increasing with larger distance and height allowances. Ultimately, the lowest false positive rate that maintained zero false negatives was 13%. This occurred with $HDR_{series}=6$ and $Ht_{max}=30ft$.

Overall results are presented in Table 3. Although adding the alignment filter described in Algorithm 3 reduces false positives and increases true negatives, it does not lead to the identification of any additional true positives.

## 5. Discussion

This article explains and evaluates methods for efficiently correlating tower observations with existing, potentially less accurate, database entries. Creating the index and uncertainty hash tables allows the rapid updating of existing entries with a common spatial hash key while significantly increasing average horizontal accuracy. Next, the article explains a way to exploit the improved accuracy of this database to determine whether a vertical structure is likely to be an electrical transmission tower. Although the categorization of transmission line towers in the Delaware DOF was mostly correct, isolated towers had to be manually removed to avoid false negatives. Removing these outliers left us with more than 99% of the original dataset.

This evaluation treated entries from a larger vertical structure database (DVOF) as updated observations. DVOF contained over ten times the number of DOF for the state of Delaware. Despite the larger number of entries, more than 30% of DOF entries were not updated. Spot checking showed that most of these DOF entries that were not updated were not contained in DVOF. This indicates that even a large database (such as DVOF) can benefit from being compared to other vertical structure listings.

This article used tower height, height uncertainty, position, position uncertainty, and date of last action to compare, contrast, and associate tower structures. In addition to these properties, vertical structure sensors (such as LiDAR or video) also collect gather geometric and radiometric information. Geometric properties, such as width or the presence of cross bars, could be another way to correlate power line transmission structures. Also, sets of transmission towers are typically made of the same material, whether that is steel, wood, or concrete. Each of these material types has a unique radiometric reflectance which could be yet another associative criteria.

The methods were evaluated against the 1063 vertical structures contained in a recent Delaware DOF. Although Delaware’s DOF offered a variety of power line structures, the state does not contain a significant amount of terrain relief. Both the database and power line inference methods rely on similar heights above ground level to consolidate duplicate entries and associate sets of transmission towers. Although the height above ground might remain similar for towers in rolling terrain, tower height could also vary to clear hills or other elevated points. Rolling terrain may also cause less uniform tower spacing, which could challenge the proposed power line inference method.

Airborne platforms that could be gathering tower locations will likely also have significant own ship position uncertainty. The platform action model that would inform the amount of uncertainty was not included in the experimental results. The uncertainty hash table construct is designed to accommodate large and overlapping horizontal uncertainties, but this ability should be verified in future work.

Future work should examine the effectiveness of the vertical structure and power line finding methods in rolling and steep terrain. Other efforts should also investigate an architecture that accepts dispersed observations, as opposed to comparing offline databases. Raw observations will contain additional details about each structure which are not included in these databases. Incorporating these radiometric and structural properties could further increase the effectiveness of associating towers when inferring power lines or comparing database entries.

## 6. Conclusions

The database correlation approach addresses critical aspects of distributed mapping for the safety-critical aviation use case. This article proposes a way of overcoming the difficult challenge of detecting power lines by considering the arrangement of associated towers which are much easier to detect. The simple and explainable process can be adapted to larger datasets supplemented with distributed observations. Comparing and correlating prior vertical obstacle databases between the wide variety of manual and automatic tower discoveries shows the potential for a comprehensive repository. This repository can be used for a variety of purposes, including reducing the risk of catastrophic obstacle collisions. The large-scale and critical nature of vertical obstacle infrastructure demands an efficient way to maintain awareness despite continuing expansion.

## Figures and Tables

**Table 1:** Distribution of horizontal accuracy for Delaware’s Digital Obstacle File.

Horizontal Accuracy (±Feet)	Quantity	Percent
20	249	23.4
50	95	8.9
100	2	0.2
250	571	53.7
500	120	11.3
1000	1	0.1
Undefined	25	2.4

**Table 2:** Comparison of existing and proposed feature mapping processes. New entries are manually (M) or automatically (A) added. For mapping that absorbs found objects, new observations overwrite (O) or supplement (S) previous feature data.

	Object Position Accuracy	New Entries Treatment	Matches Objects Arrangement	Matches Individual Objects	Object Correlation
DOF, GeoPackage [12]	✓	M	-	✓	S
2D projection [6,7]	-	A	-	✓	O
Quantization [4,5]	-	-	✓	-	-
Predictive mapping [11,16]	-	A	-	-	O
Point cloud matching [14]	-	-	✓	-	-
Proposed database updating method	✓	A	-	✓	S

**Table 3:** Optimum setting for power line inference. HDR: height–distance ratio, AHB: additional height buffer, $\theta_{align}$: minimum angle for alignment, $Ht_{max}$: maximum height difference among aligned tower series, $HDR_{series}$: height–distance ratio for aligned tower series, TN: true negative, TP: true positive, FN: false negative (excludes previously described examples of isolated transmission towers), FP: false positive.

HDR, ν	AHB, α (ft)	$\theta_{\mathrm{align}}$ (deg)	${\mathrm{Ht}}_{\max}$ (ft)	${\mathrm{HDR}}_{\mathrm{series}}$	TN	TP	FN	FP	FP (%)
9	85	-	-	-	471	408	0	155	14.99
		4	30	6	492	408	0	134	12.96

## Data Availability

The publicly available Digital Obstacle File (DOF) was analyzed in this study. Restrictions apply to the availability of Digital Vertical Obstruction Files (DVOF). DVOF was obtained from the NGA’s Aero Browser and is available with the permission of the National Geospatial-Intelligence Agency. Satellite imagery in figures is from Google Earth.

## Abbreviations

The following abbreviations are used in this manuscript:

AAM	Advanced Aerial Mobility
AGL	Above Ground Level
AHB	Additional Height Buffer
CFIT	Controlled Flight into Terrain
DOF	Digital Obstacle File
DVOF	Digital Vertical Obstruction File
FAA	Federal Aviation Administration
FN	False Negative
FP	False Positive
HDR	Height–Distance Ratio
IHT	Index Hash Table
LiDAR	Light Detection and Ranging
NGA	National Geospatial-Intelligence Agency
TN	True Negative
TP	True Positive
UAS	Uncrewed Aircraft Systems
UHT	Uncertainty Hash Table
WGS	World Geodetic System
